# Developing a high-resolution gridded rainfall product for Bangladesh during 1901–2018

**DOI:** 10.1038/s41597-022-01568-z

**Published:** 2022-08-03

**Authors:** Ashraf Dewan, Shamsuddin Shahid, Md. Hanif Bhuian, Shaikh M. Jobayed Hossain, Mohamed Salem Nashwan, Eun-Sung Chung, Quazi K. Hassan, Md Asaduzzaman

**Affiliations:** 1 Spatial Sciences discipline, Curtin University, Bentley 6102, Perth, Western Australia; 2 School of Civil Engineering, Faculty of Engineering, Universiti Teknologi Malaysia (UTM), 81310 Johor Bahru, Malaysia; 3 Department of Geography and Environment, Jagannath University, Dhaka, 1100 Bangladesh; 4 Meiji University, Chiyoda city, Tokyo, 101-8301 Japan; 5 Construction and Building Engineering Department, College of Engineering and Technology, Arab Academy for Science, Technology and Maritime Transport (AASTMT), 2033 Cairo, Egypt; 6 Department of Civil Engineering, Seoul National University of Science and Technology, Nowon-gu, 01811 Seoul, South Korea; 7 Department of Geomatics Engineering, University of Calgary, 2500 University Drive NW, T2N 1N4 Calgary, Canada; 8 Department of Engineering, School of Digital, Technologies and Arts, Staffordshire University, Stoke-on-Trent, UK

**Keywords:** Hydrology, Hydrology

## Abstract

A high-resolution (1 km × 1 km) monthly gridded rainfall data product during 1901–2018, named Bangladesh Gridded Rainfall (BDGR), was developed in this study. *In-situ* rainfall observations retrieved from a number of sources, including national organizations and undigitized data from the colonial era, were used. Leave-one-out cross-validation was used to assess product’s ability to capture spatial and temporal variability. The results revealed spatial variability of the percentage bias (PBIAS) in the range of −2 to 2%, normalized root mean square error (NRMSE) <20%, and correlation coefficient (R^2^) >0.88 at most of the locations. The temporal variability in mean PBIAS for 1901–2018 was in the range of −4.5 to 4.3%, NRMSE between 9 and 19% and R^2^ in the range of 0.87 to 0.95. The BDGR also showed its capability in replicating temporal patterns and trends of observed rainfall with greater accuracy. The product can provide reliable insights regarding various hydrometeorological issues, including historical floods, droughts, and groundwater recharge for a well-recognized global climate hotspot, Bangladesh.

## Abstract (summary)

**Table:** 

Measurement(s)	water-based rainfall
Technology Type(s)	weather station

## Background

Located in a tropical monsoonal climatic region, Bangladesh receives nearly 2200 mm of rainfall every year, which supports agriculture, the environment and livelihood activities since time immemorial^1–3^. However, the country is susceptible to hydrometeorological hazards due to its flat topography and high rainfall seasonality^4,5^. For instance, nearly 70% of total rainfall occurs in the monsoon season (June to September), and <3% takes place in the dry season (December to February)^6^. Any change in rainfall patterns in terms of deficit/surplus or even a subtle shift can lead to climatic extremes such as drought and floods. Ozaki^7^ showed that the country experiences economic damage of US$ 2.2 billion, equivalent to 1.5% of gross domestic product (GDP) during an abnormal flood year. It experiences a reduction in crop production of 20 to 30% in a drought year^8^. Nearly 40% of total crops in the country are rain-fed, and the rest depend on groundwater irrigation^9^, meaning that rainfall variability, especially monsoonal amount (1500 mm), severely affects rain-fed agriculture, which has implications for food security due to its large population. It is also a primary source of groundwater replenishment. For instance, a moderate rainfall deficit causes a decline in groundwater in subsequent years^10^, causing an increase in irrigation costs, reducing farmers’ profit, and overwhelming a vast majority of the rural population^11^. Since large river networks of the country are fed by Himalayan snowmelt and local rainfall, high rainfall evidently affects riverbank erosion^12^, while low rainfall reduces freshwater flow towards coastal areas, causing salinity ingress further inland^13^ and affecting the livelihoods of millions.

With the increased concern of anthropogenic climate change, the demand for high-resolution gridded datasets of climate variables such as rainfall has increased over the last few decades^14^. Consequently, a number of daily and monthly gridded products have been developed at global^15–17^, regional^14,18,19^ and national scales^20–23^ with spatial resolutions varying from 0.01 to 1.0°. Several interpolation methods, such as kriging^24^, angular distance weighting^25^, minimum surface curvature^26^, thin plate splines^27^, and inverse distance weighting^28^, are utilized. Although global and regional gridded rainfall products have enhanced our knowledge of extreme events, determining their space–time variability and pattern requires high-resolution datasets^21^. Thus, existing global- or regional-scale products may not support the evaluation of local-scale extreme events and spatiotemporal variability^22^. Fine spatial resolution (~0.01°) is, therefore, a prerequisite to capture environmental variability^17^. Furthermore, high-resolution data can be instrumental to examine climate–impact studies on an area^23^.

While increases in near-surface air temperature have been well documented for Bangladesh^6^, lack of long–term high–resolution rainfall data was an important constraint to determine changes in spatiotemporal rainfall pattern. A dense rainfall monitoring network is needed to assess the spatiotemporal variability of hydrometeorological hazards for developing mitigation measures. It is also important to examine how increased anthropogenic activities resulting from rapid urban expansion, forest loss associated with land use/land cover changes, alteration of fluvial morphology and ever-increasing populations influence regional climate. However, such studies in the country are few and far between. The unavailability of long-term rainfall data is thought to have contributed to the nonexistence of a detailed regional-scale analysis of climatic conditions such as the spatiotemporal pattern of rainfall variability.

The Bangladesh Meteorological Department (BMD) has 42 rainfall observation stations, but their distribution is not uniform across the country (http://live.bmd.gov.bd/). The majority of these stations were installed in the 1960s, and therefore, data are available only since 1960s. The number of stations is also far below (one station per 3428 km^2^) the recommended number by the World Meteorological Organization (WMO), which is one station per 10−20 km^2^ for climatic studies^29^. However, almost all studies employ BMD data and appear to provide coarse spatial detail regarding rainfall climatology and their trend for the country. Compared to BMD, the Bangladesh Water Development Board (BWDB) maintains an extensive rainfall monitoring station. It currently has 293 rain gauges over 130,170 km^2^ of land area (www.ffwc.gov.bd). Combining rainfall records of BWBD and BMD provides good coverage over the country, but the inhomogeneous distribution of stations and unavailable/missing data during the last century inhibit long-term regional assessment.

Then, East Bengal (currently Bangladesh) used to experience frequent floods, droughts and cyclones; thus, rainfall data were indispensable to better prepare against these hazards and to reduce the loss of lives and property during the British Raj. The Public Works Department (PWD) during the colonial government therefore installed rainfall monitoring stations in the region. The monthly rainfall records of those stations were later published by the East Pakistan (present Bangladesh) Water and Power Development Authority in two separate volumes of water supply papers (WSP)^30,31^. The undigitized rainfall information from 1901 to 1959 is of high value and is a reliable source to examine the rainfall distribution of the region since 1901. However, rainfall data during that period have not been utilized in any of the studies due to limited access and/or their unavailability in digital form. This work was motivated to harness the power of digital technologies by which more than a hundred years of rainfall data were encoded to generate a high-resolution gridded product.

It is obviously a major challenge to develop a rainfall product, especially when the data were recorded by different instruments and maintained by various origins. To make them useable, proper quality control and homogenization are essential. Furthermore, selecting a suitable interpolation method to generate gridded dataset with station data is also important. This study presented the development of a fine-resolution rainfall product by using multiple sources of data. It is expected that the product would be of great value to advance climatic and hydrological studies of Bangladesh, a global hotspot of anthropogenic climate change.

### Geography and precipitation climatology of Bangladesh

**Fig. 1:**
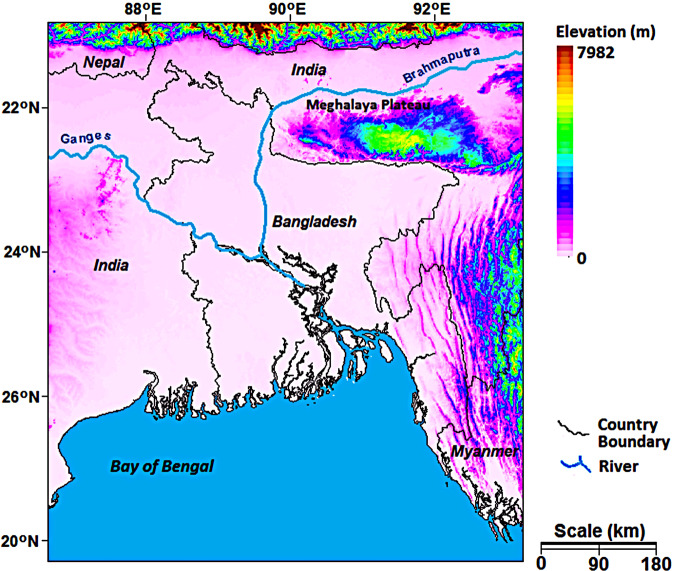
Location and topography of Bangladesh. Bangladesh, located between 20°34′–26°38′ N and 88°01′–92°41′ E, covers an area of 147,570 km^2^. The elevation of the country varies from 0 m in the south to approximately 110 m in the extreme north. Low hills and highlands in the southeast and northeast cover only 10% of the total land.

Bangladesh, located between 20°34′–26°38′ N and 88°01′–92°41′ E, covers an area of 147,570 km^2^. Approximately 90% of the land is a deltaic plain, known as the lower Gangetic plain. The elevation of the country varies from 0 m in the south to approximately 110 m in the extreme north^32^. Low hills and highlands in the southeast and northeast cover only 10% of the total land. The maximum elevation of the hilly regions reaches 1010 m. The geographical position of the country in the three mighty rivers, viz., the Ganges, Brahmaputra and Meghna (Fig. 1), made it highly prone to recurrent floods.

Rainfall of the country is controlled by differential heating of land and sea surfaces. Indian landmass heated rapidly compared to its surrounding oceans, introducing a strong thermal contrast between land and sea^33^. Therefore, air flows from the oceans towards heated land. The moist air from the sea enters Bangladesh from the south and causes high monsoon rainfall from June to September. A reveres situation occurs during the winter season from December to February, and air flows from land to the sea^34,35^. The air flowing over the landmass is dry and thus experiences almost no rainfall during winter. Between summer and winter, there are two transitional periods, post-monsoon (October–December) and premonsoon (March–May). While the former is relatively cool, the latter is hot with occasional downpour.

The monsoon of Bangladesh flows in two branches, one of which strikes western India, and the other travels up to the Bay of Bengal before entering Bangladesh via the southeast. The monsoon from the Bay of Bengal crosses the plain to the north and northeast before being turned to the west and northwest by the foothills of the Himalayas^6,36^. As monsoon air moves farther inland, its moisture content decreases, resulting in decreased rainfall towards the northwest and west of the country^37^. However, the additional uplifting effect of the Meghalaya Plateau increases rainfall to the northeast. The premonsoon is a transitional season between the northerly circulation of winter and the southerly circulation of the monsoon. Thunderstorms are a major source of premonsoon rainfall^38,39^. The precipitation mechanism indicates that topography and distance to coast can also have a large influence on its spatial distribution and seasonality.

### Data acquisition

Monthly rainfall records were acquired from three sources. They were:

(i) BMD: daily rainfall records of 33 stations were collected. Data for nearly 52% stations were available after the 1960s, although only three stations had data since 1901.
(ii) BWDB: daily rainfall records of 293 stations between 1961 and 2018 were obtained and utilized in this work. Note that data were available for 1961–2018 at 52% stations, while others provided data during different temporal windows of 1961–2018.
(iii) WSP: The WSP rainfall records were available for 124 locations from 1901 to 1959^30^. These records were available in printed form, and the digitizing process includes editing and proofreading before publication, as in other government gazetteer reports. They were encoded with caution and cross-checked several times to ensure high fidelity. However, only 45% of stations contain records from 1901 to 1959. The rest of the stations have data for different temporal resolutions of 1901–1959.

**Fig. 2:**
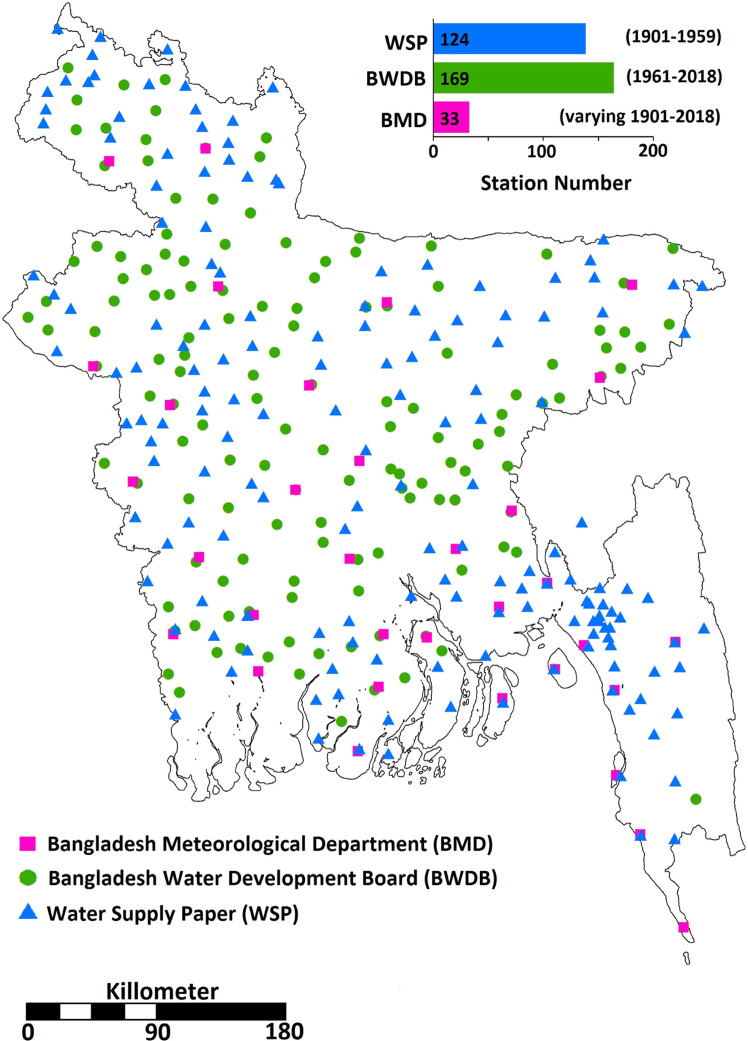
Distribution of rainfall stations and their records. Monthly rainfall records were acquired from three sources: 33 stations from BMD for different periods, 293 stations from BWDB for different windows between 1961 and 2018, 124 stations from WSP for different windows between 1901 and 1959. Attributes of 124 stations (e.g., name and locations) are common for both BWDB and WSP, meaning that 124 stations have data for different temporal windows from 1901 to 2018. Compilation of a database using rainfall data from all sources provides records of a total of 326 stations crisscrossing over Bangladesh.

Attributes of 124 stations (e.g., name and locations) are common for both BWDB and WSP, meaning that 124 stations have data for different temporal windows of 1901 to 2018. Compilation of a database using rainfall data from all sources provides records of a total of 326 stations, crisscrossing over Bangladesh (Fig. 2). Furthermore, a more or less homogeneous distribution of rain gauges can be seen (Fig. 2), except for southwestern dense and southeastern forested mountainous regions.

Daily data were aggregated to generate monthly rainfall time series. The general rules in aggregating daily rainfall to monthly rainfall differ for monsoon and nonmonsoon months. The monsoon month’s rainfall was considered unavailable if rainfall in a single day was missing, while rainfall of a nonmonsoon month was considered missing if records for three consecutive days were missing^40^. WSP data were available only at the monthly scale, whereas BMD and BWBD had daily data. To bring them in a common temporal resolution, data were aggregated to monthly time series using the rule–based procedures noted above.

Stations having observations for at least 25 years with random missing values were included in the present study. Short-term records (<25 years) were removed to avoid noise. Few duplicates were identified due to changes in station names or locations in the government gazetteers. In addition, few stations were adjacent to each other. This was due to a slight change in the installation locations after decommissioning the previous station. Duplicates were removed, and stations in close proximity (<500 m) were averaged. In the case of a locational change, the station was given a separate name. Thus, the new and old stations were considered as a single station. The station’s new location is used for processing data recorded after locational shift.

**Fig. 3:**
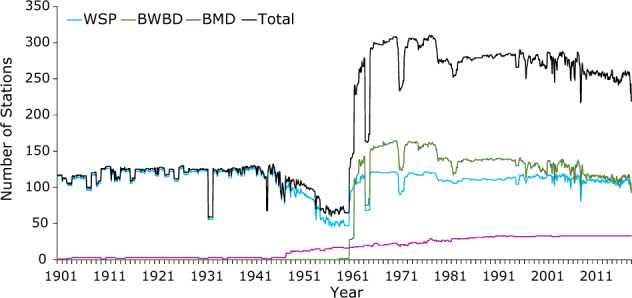
Availability of data from different sources. Number of available meteorological stations per year from different sources during 1901–2018.

**Fig. 4:**
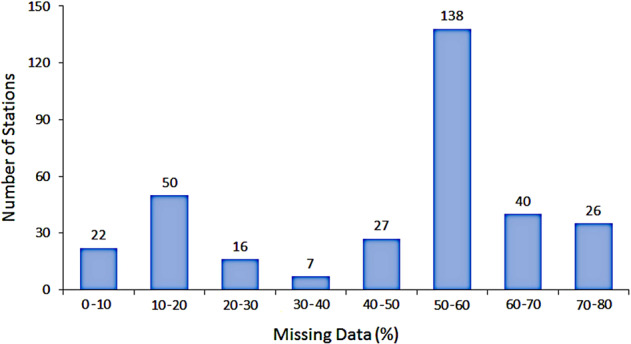
Missing records (%) during 1901–2018. The number of stations with different percentages of missing data during 1901–2018. The highest number of stations (138) had missing rainfall in the range of 50 to 60%, and 66 stations had more than 60% missing records.

Figure 3 shows the availability of data from different sources during 1901–2018. Missing data over the study period range between 3.2% and 76.1% for different stations. The percent of missing records at different stations is shown in Supplementary Figure S1. The number of stations with different percentages of missing data during 1901–2018 is shown in Fig. 4, which shows that the highest number of stations (138) having missing rainfall is in the range of 50 to 60%, and 66 stations had more than 60% missing records.

The Precipitation Bias CORrection (PBCOR)^41^ data was used to evaluate the rainfall climatology of the BDGR product. PBCOR is a global high-resolution (0.05°) precipitation climatology bias-corrected using streamflow. It consists of bias-corrected precipitation climatologies of WorldClim V2 for the period 1970–2000^42^, Climatologies at High Resolution for the Earth’s Land Surface Areas (CHELSA V1.2) for 1979–2013^43^, and Climate Hazards Group Precipitation Climatology (CHPclim V1) for 1980–2009^44^. These WorldClim, CHELSA and CHPclim climatologies underestimate precipitation in the broader Himalayas region, including some parts of Bangladesh^45^. PBCOR corrected this bias to develop PBCOR WorldClim, PBCOR CHELSA and PBCOR CHPclim to provide better estimates of the precipitation climatologies.

### Methodology

#### Gap filling

In this study, multivariate imputation by chained equations (MICE) was used to fill in missing rainfall data. The MICE was implemented considering missing data at all stations in the study area. The MICE method estimates missing values of rainfall data from a station using rainfall data from its nearby stations. In the first step, the MICE replaces missing values for each station of the set with the mean rainfall of the station as initial estimates. It then sets back the first estimated missing value as missing while keeping the other imputed values as original values and regresses them. The rest of the imputed missing values were regressed in the same way. The regressed values were then subtracted from the initial set of data, which gives errors for missing value estimates. Further steps generate errors in the same way by setting imputed values as missing one-by-one, regressing them and subtracting them from the previously generated sets of data. The process was repeated until the change in estimated error in predicting missing data is not significant. In the present study, the process was repeated five times, as no noticeable improvement in the error matrix was found when multiple imputations were more than five times. The preliminary analysis of filling in missing data of a station was performed using data from 4 to 10 nearby stations. The results showed no improvement in prediction error if more than six nearby stations were used. Therefore, rainfall data from six nearby stations were used to fill in the missing data of each station.

The mice package^46^ of R program was used in this study to fill in missing data. Different classical models, for instance, linear regression, logistic, Poisson, polytomous regression, and random forest, can be used in MICE for imputation model development. This study evaluated the performance of different methods available in the mice package in R and found the best performance using ‘random forest’ for the number of trees (ntree) equal to fifty.

The performance of MICE models was evaluated for filling both continuous and random missing data. The results showed poor performance of the method in filling in continuous missing data for more than four years. It should be noted that no data were available for 184 stations before 1961. Some of these stations also contain continuous missing data for four years after 1961. Rainfall data with random missing values for these stations were available for 29 to 56 years. Filling nearly 60 years of missing rainfall data with the model developed using 29 to 56 years of data would lead to large uncertainty. Therefore, these 184 stations’ data were not filled during 1901‒1960. However, random missing data from these stations after 1961 were filled. The missing data at the remaining 142 stations during 1901–2018 were between 3.2 and 54.4%. Continuous missing rainfall data at 70 out of these 142 were noticed for 4 to 26 years, which were also not filled. After leaving the continuous missing rainfall for more than four years, the random missing rainfall data at all stations ranged from 3.2% to 28.8%. However, at nearly 90% of the stations, the random missing data were below 20%. These random missing data were filled with the MICE model.

### Homogenization of the rainfall series

Longer period rainfall series are significantly affected by inhomogeneities because of upgrading the instruments, relocation of stations and measurement error. These factors often result in unexpected jumps or shifts, reflecting outliers in the data series. Therefore, the detection of inhomogeneities and homogenization of climate data is essential^47,48^. In this study, Climatol^49^, available in the statistical package R, was used to homogenize the data series. Coll, *et al*.^48^ compared the break frequency results of different homogenization methods and reported superior performance of Climatol than the other methods. Skrynyk, *et al*.^50^ evaluated the uncertainty associated with Climatol’s adjustment algorithm and reported a better capacity of Climatol to remove systematic errors related to jumps in the means. Domonkos, *et al*.^51^ also showed Climatol to be effective for homogenizing climate data. Therefore, it has been widely used as an effective climate data homogenization tool in recent years^52,53^. Details of Climatol can be found in Guijarro^54^.

Climatol uses a standard normal homogeneity test (SNHT) both on overlapping windows and on the whole rainfall series to detect inhomogeneities. It first uses the whole series to detect the break and then splits the series based on breakpoints to reveal any other breaks in its subseries. Multiple checks provided a better capability of Climatol to manage multiple inhomogeneities. Finally, it fills all missing values using the weighted ratio of data from neighbouring stations to make the series homogeneous by using the reduced major axis model^55^, a kind of orthogonal regression, $\widehat{{y}_{i}}={x}_{i}$, where *x*_*i*_ is the mean (or the weighted mean) rainfall (mm) of nearby stations, and $\widehat{{y}_{i}}$ is the data (mm) containing missing values. Both *x*_*i*_ and $\widehat{{y}_{i}}$ are standardized based on available data before developing the regression method.

It should be noted that Climatol fills the missing data with spatial interpolation. However, these filled values were not used in generating BDGR data. Only the random missing data were filled using MICE before homogenization using Climatol. Missing data were filled with Climatol and replaced with NA before using the data for generating BDGR and cross validation.

### Product development procedure

**Fig. 5:**
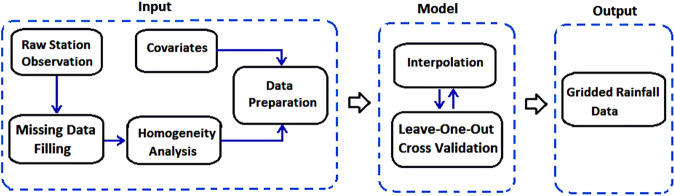
Flowchart showing the procedures. Flowchart showing the procedures used for generating the BDGR product. Leave-one-out cross-validation (LOOCV) was employed, where only a single observation was used for validation and the remaining observation was used for model training. The process was repeated for N (number of observations) time to cover all observations as a validation sample.

Leave-one-out cross-validation (LOOCV) was employed, where only a single observation was used for validation and the remaining observation was used for model training (Fig. 5). This process was repeated for N (number of observations) time to cover all observations as a validation sample. The LOOCV approach required a large computation time. However, it provided error estimation for each station, thus, quantifying errors both spatially and temporarily.

The process was repeated for all months for all years between 1901 and 2018. In each case, outliers or abnormal values were examined using residuals. Nearby station data were checked to examine whether there are any outliers or abnormal values. This was done for every station considered in this work. If the difference in rainfall from all nearby stations was greater than 50% of the mean rainfall of a station, the data are outliers, possibly caused by mechanical faults or human error, and were considered missing.

#### Thin-plate spline (TPS) interpolation

Observed rainfall data were interpolated using the TPS method^56^. It is a robust technique and is used for developing gridded air temperatures of China^57^, ‘climate surfaces’ of global land areas^17,42^, and high–resolution daily precipitation datasets of Europe^58^. Details about TSP can be found in Hutchinson and Xu^56^. In TPS, N rainfall data are used to fit the spline model, and *z*_i_ (mm) takes the following formula^56^:

1 $${z}_{i}=f\left({x}_{i}\right)+{b}^{T}{y}_{i}+{e}_{i}$$

where i = 1 … N, *f* is a smooth function of the spline independent variables *x*_i_ (mm), *b* is a vector of linear coefficients for independent covariates, and *y*_i_ and *e*_i_ are independent, zero-mean error terms^56^. The models were developed using different combinations of covariates, and the model producing the least error was finally used for interpolation.

Rainfall data for each month of all years between 1901 and 2018 were interpolated separately to generate gridded rainfall surfaces for individual months. All interpolated surfaces are then integrated to produce gridded monthly rainfall time series for the study period over Bangladesh.

### Error metrics

The BDGR product, generated through the TPS model, was validated using the normalized root mean squared error (NRMSE) in %, bias (PBIAS) in %, coefficient of determination (R^2^), Kling-Gupta efficiency (KGE), Willmott’s modified coefficient of agreement (MD) and Perkins skill score (PSS). Besides, the ratio of standard deviation (rSD) was used to assess the missing rainfall data estimation model. The equation of the metrics, their range and optimum values are provided in Table 1. The NRMSE and PBIAS were widely used metrics to estimate errors in the data product, while R^2^ is a universal metric for showing an association between two time series, i.e., observed versus gridded data. The KGE was an integrated metric that measures association, similarity in the variance and mean between two variables^59^. The MD provides appropriate weight to error and differences between two variables for measuring agreement^60^. Therefore, it provided a better estimate of association in the case of extreme values. It was expected that gridded data should replicate the probability distribution of observed rainfall. Therefore, PSS was used, which measures how the probability distribution function (PDF) of two variables matches each other^61^. In Table 1, *r* is Pearson’s correlation; *μ* and *σ* represent the mean and standard deviation of gridded (*S*) and observed (*O*) rainfall (mm); N is the sample size; and *f*_*o*_ and *f*_*s*_ are PDFs of the observed and gridded rainfall (mm).

**Table 1:** Description of statistical metrics used for evaluating data product.

Metric Formula	Range	Unit	Optimal Value
$NRMSE=100\ast \frac{\sqrt{\frac{1}{N}\ast {\sum }_{i=1}^{N}{\left({S}_{i}-{O}_{i}\right)}^{2}}}{{\mu }_{o}}$	0–∞	%	0
$PBIAS=100\ast \frac{{\sum }_{i=1}^{N}\left({S}_{i}-{O}_{i}\right)}{{O}_{i}}$	−∞–∞	%	0
${R}^{2}=1-\frac{{\sum }_{i=1}^{n}{\left({O}_{i}-{S}_{i}\right)}^{2}}{{\sum }_{i=1}^{n}{\left({O}_{i}-{\mu }_{o}\right)}^{2}}$	0–1	—	1
$md=1-\frac{{\sum }_{i=1}^{n}\left({O}_{i}-{S}_{i}\right)}{{\sum }_{i=1}^{n}\left(\left|{S}_{i}-{\mu }_{o}\right|+\left|{O}_{i}-{\mu }_{o}\right|\right)}$	0–1	—	1
$KGE=1-\sqrt{{(r-1)}^{2}+{\left(\frac{{\mu }_{s}}{{\mu }_{o}}-1\right)}^{2}+{\left(\frac{{\sigma }_{s}/{\mu }_{s}}{{\sigma }_{o}/{\mu }_{o}}-1\right)}^{2}}$	−1–∞	—	−1
$rSD={\sigma }_{o}/{\sigma }_{s}$	−∞–∞	—	1
$SS=\mathop{\sum }\limits_{i=1}^{n}\,\min \left({f}_{s},{f}_{o}\right)$	0–1	—	1

## Technical Validation

### Performance of gap filling

**Fig. 6:**
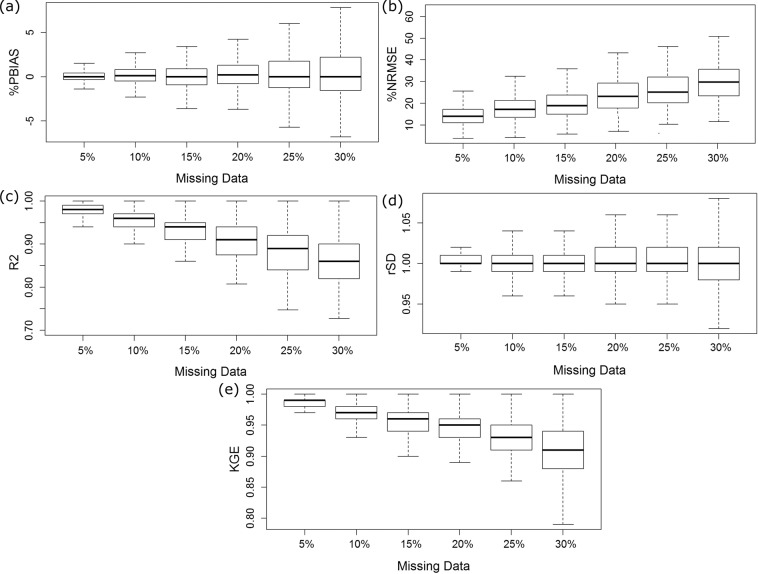
Performance in filling missing values. Performance in estimating missing rainfall values in terms of: (**a**) bias in % (PBIAS); (**b**) normalized root mean squared error (NRMSE) in %,; (**c**) coefficient of determination (R^2^); (**d**) ratio standard deviation (rSD); and (**e**) Kling-Gupta efficiency (KGE).

The MICE models were developed by randomly selecting 70 to 95% of the available data and then validating the remaining 5 to 30% per data at each station. The model’s performance was evaluated using statistical metrics at all stations and is presented in Fig. 6. The performance was evaluated for 5 to 30% of missing data to show the robustness of the MICE model developed in this study in filling different amounts of missing values. The results showed that the mean bias in 5 to 30% missing rainfall was 0.1 to 0.9, and the NRMSE was between 14.5 and 29.1%. For all cases, the mean R^2^ was above 0.86, rSD was near 1 and KGE was above 0.92. In particular, the metric values showed that the performance of the MICE model in missing rainfall of less than 20% was near ideal values. This indicates perfect filling of random missing values of less than 20% at nearly 90% of stations.

### Homogenization of rainfall series

**Fig. 7:**
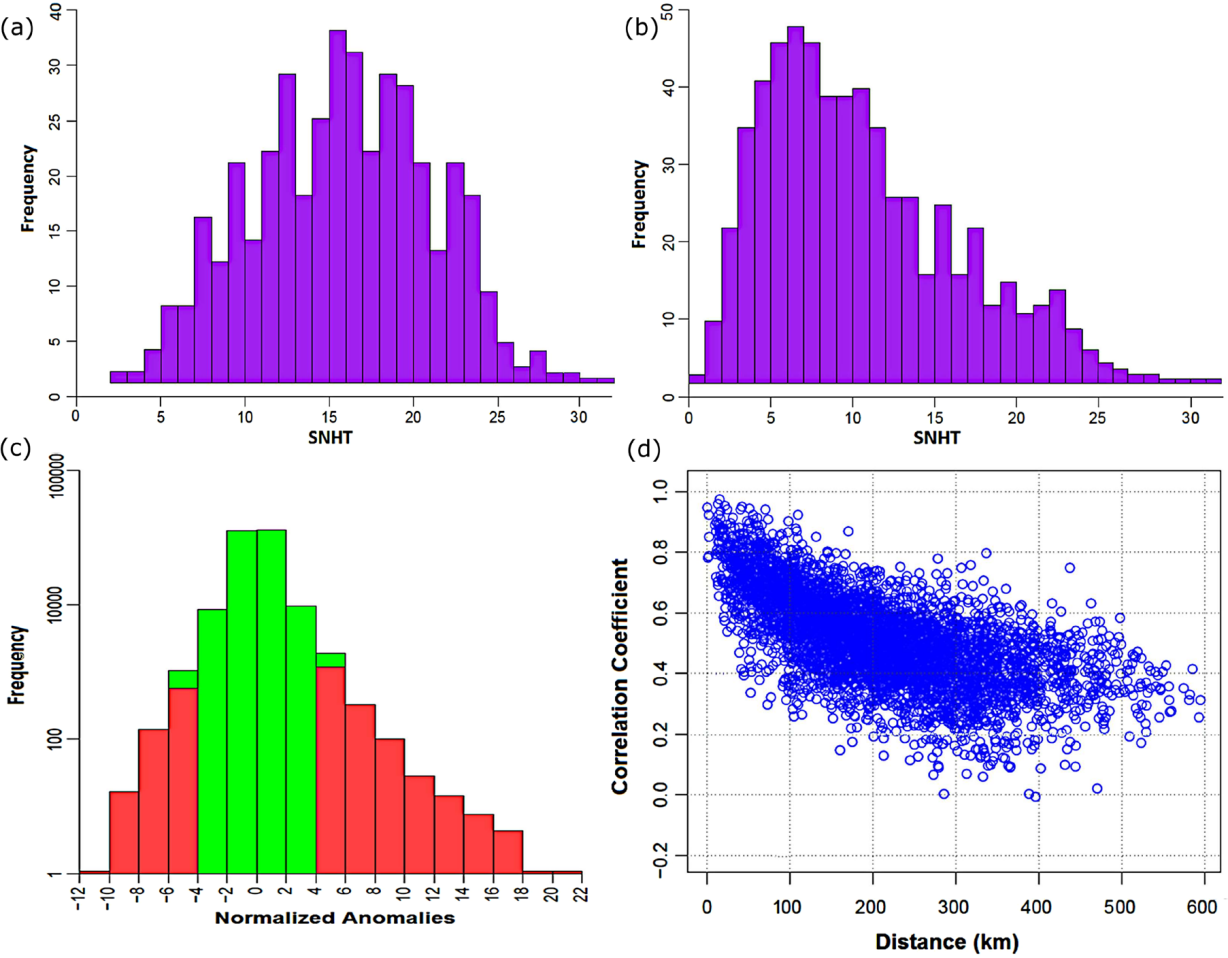
Standard normal homogeneity test (SNHT) results. Optimum SNHT values were determined through visualization of histograms. (**a**,**b**) shows the varying maximum SNHT values of 326 stations for overlapping windows and whole series, respectively; (**c**) normalized anomalies during exploratory run; and (**d**) correlogram of the entire rainfall series.

Data homogenization requires determination of optimum SNHT values for overlapping windows and for the entire series. In this study, SNHT values were determined through visualization of histograms. Figure 7(a,b) shows the varying SNHT values of 326 stations. Careful observation of the histograms reveals an apparent minimum SNHT value after 28. Therefore, the SNHT value for both the overlapping windows and the whole series was 28.

Figure 7(c) shows a histogram of normalized anomalies used for outlier detection. Anomalous values beyond ±5 standard deviations (SDs) were considered outliers. A total of 972 outliers were observed. All outliers were manually checked and compared with neighbouring station data. Few outliers resulted from data entry; however, most of the values seemed to have legitimate values. After correcting outliers associated with data entry, no values are found beyond ±18 SDs. Therefore, a threshold of ±18 SDs was chosen to incorporate all extremes.

Figure 7(d) shows a correlogram of monthly rainfall between stations. The correlation for stations within a 50 km radius was >0.75 and decreases rapidly after 100 km. A high correlation with nearby stations and gradually a low relationship with increasing distance was expected due to synoptic–scale moist air circulation that influences rainfall in Bangladesh. Overall, the correlogram indicates good quality of the observed data.

Climatol detected a total of 132 breaks in the whole rainfall series (i.e., 326). Some of the breaks can be explained with the aid of literature. For example, Shahid^39^ showed abrupt changes in rainfall at some stations between 1973 and 1975. However, most of them could not be explained due to unavailability of metadata. All the breaks were visually inspected and compared with nearby stations. Highly abnormal breaks were discarded, while others were accepted. This study finally detected homogeneity of 322 out of 326 time series. These 322 series were finally employed to develop a high-resolution gridded product for Bangladesh.

### Development of gridded product

The initial assessment was conducted to select the covariates and interpolation method. Data from 80% of stations were used for model development and the rest for validation in this stage. The present study evaluated similar covariates (topography and distance to coast) used in developing WorldClim^42^ data to improve the performance of interpolation. Previous studies^33,39^ indicated the influence of topography and distance to coast on rainfall in the country. High rainfall is generally recorded at elevated locations^40^. In addition, air moisture usually decreases from the coast to inland^62^. These two covariates, i.e., elevation and distance to coast, are accounted for in this study. The TPS models of the work were therefore developed: (a) without covariates; (b) each of the covariates included separately; and (c) employing both covariates simultaneously. In addition, models are developed considering covariates as independent and linear. Since there is a non–linear effect of elevation and distance to coast on rainfall in the country, data are transformed using log and square root prior to inclusion in the TPS models. Prediction of multiple models developed with different combinations of covariates, considering their linear and nonlinear effects. However, the results show no improvement, and therefore, the model was developed without any covariates.

Kriging was one of the most efficient and widely used interpolation methods^25^. Therefore, the performance of TPS was compared with kriging to justify the use of TPS in generating gridded products. A similar process as the TPS was followed to validate the output of the kriging model. The results are presented in Figure S2 (supplementary materials), which show a nearly 2 to 10% improvement in the median of the evaluation metrics using the TPS method.

### Performance of gridding method

**Fig. 8:**
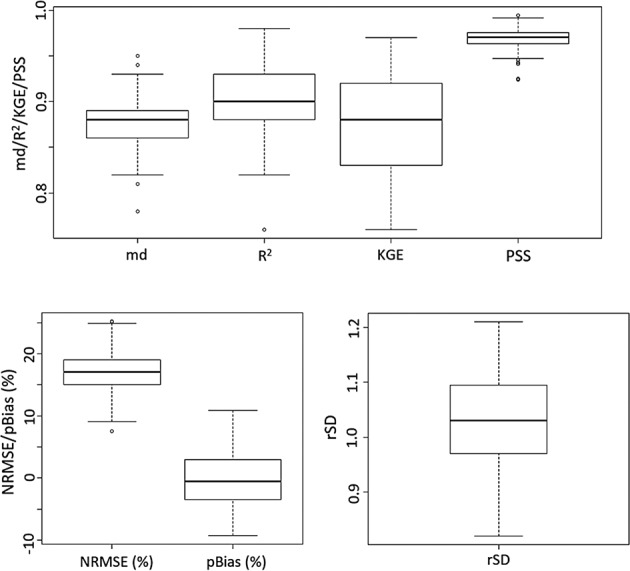
Performance of the thin-plate spline model. Performance of the thin-plate spline model in reconstructing observed rainfall during 1901–2018. The boxplots show the model’s performance for the whole period (1901–2018), based on normalized root mean squared error (NRMSE) in %, bias (PBIAS) in %, coefficient of determination (R^2^), Kling-Gupta efficiency (KGE), Willmott’s modified coefficient of agreement (MD) and Perkins skill score (PSS).

The performance of TPS models without considering any covariates at all stations is presented in Fig. 8. The model’s performance for the whole period (1901–2018), based on NRMSE, PBIAS, R^2^, KGE, MD and PSS, is shown in Fig. 8(a). The medians of KGE, MD and PSS were 0.87, 0.90 and 0.98, respectively. The minimum values of the indices were 0.66, 0.83 and 0.96. The ranges of NRMSE and PBAIS were 9.3 to 20.2 and −9.7 to 11.7, and their medians were 17.2 and −0.9%, respectively. The median of rSD was 1.04. The results indicate a good performance of the TPS model in reconstructing rainfall time series of all rainfall stations (i.e., 322). The product was also evaluated by checking any abnormality in the generated surfaces compared to known monthly rainfall patterns or small circular patterns by potential outliers. No abnormal rainfall pattern was noticed for any month examined, indicating good homogenization of the series. LOOCV was used to estimate the performance of interpolated rainfall at each station to show the spatial and temporal variability of the metrics.

### Performance of BDGR

#### Statistical evaluation

**Fig. 9:**
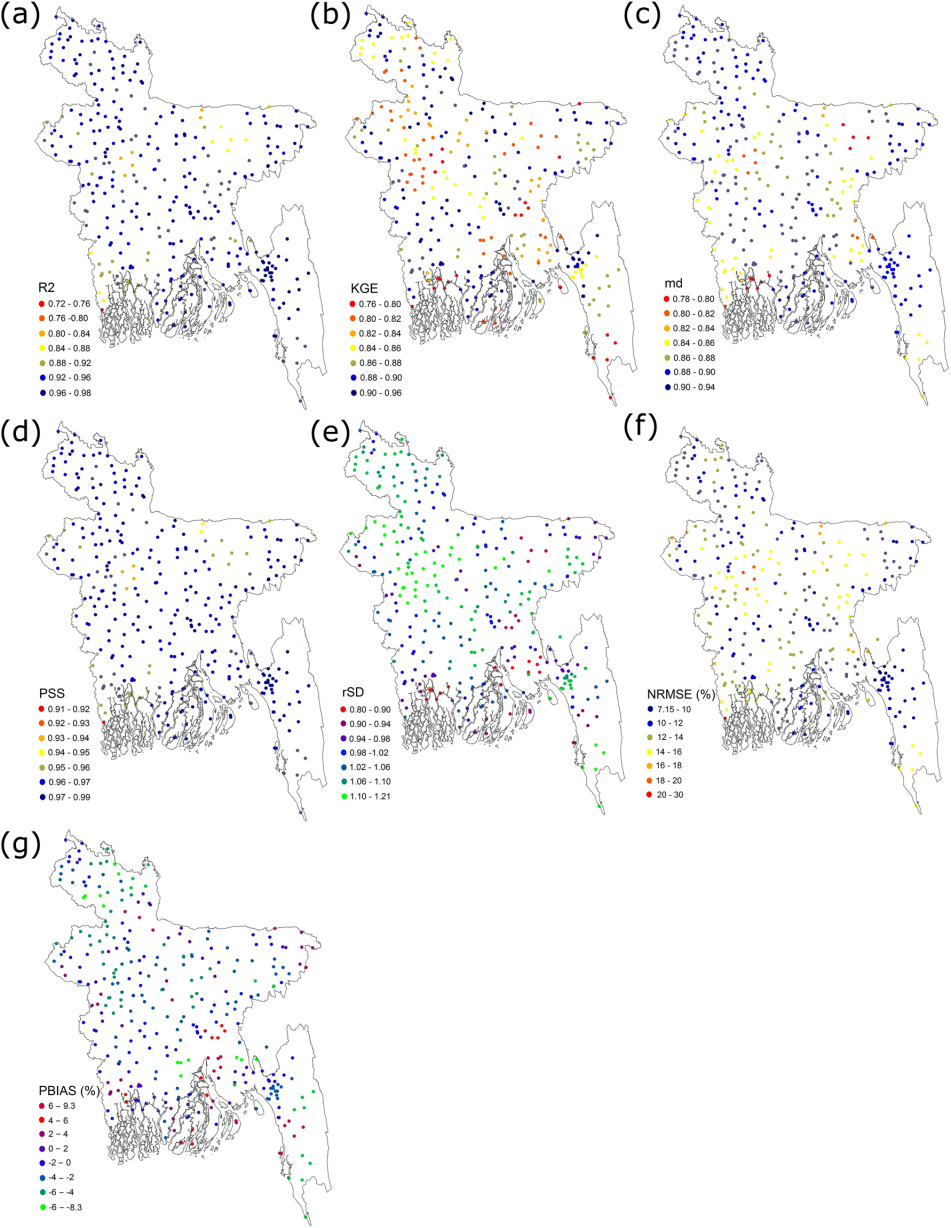
The spatial distribution of BDGR performance. The spatial distribution of BDGR performance in terms of (**a**) coefficient of determination (R^2^); (**b**) Kling-Gupta efficiency (KGE); (**c**) Willmott’s modified coefficient of agreement (MD); (**d**) Perkins skill score (PSS); (**e**) ratio of standard deviation (rSD); (**f**) root mean squared error (NRMSE) in %; and (**g**) bias (PBIAS) in %.

The LOOCV approach provided error estimation for each station, thus quantifying errors in estimated rainfall both spatially and temporarily. The performance of rainfall at each station was used to prepare the maps to show the spatial distribution of the performance metrics of BDGR (Fig. 9). The association metrics, R^2^ and MD, revealed high correlations of observed and interpolated rainfall time series at most stations. The R^2^ values were more than 0.88 at 79% of the stations and between 0.72 and 0.76 at only 2.6% of the stations. The MD was more than 0.86 at 88% of stations and between 0.79 and 0.82 at only three stations. The KGE was more than 0.8 at 98.5% of the stations. The PSS was above 0.96 at 93% of stations, while rSD was between 0.94 and 1.06 at 83% of stations. Among the two error metrics, NRMSE showed less than 14% error at 57% of the stations while between 20 and 30% only at 7% of stations. The PBIAS was between −2 and 2 at 78% of stations. It was out of the range of −6 to 6 only at nearly 8% of stations. The spatial distribution of the metrics showed a random distribution of high and low values over most of the country, except for the southwest and southeast corners. This was due to the low density of observation data at these locations. The northwest region is covered by dense forest, while the northwest corner is mountainous and less populated.

**Fig. 10:**
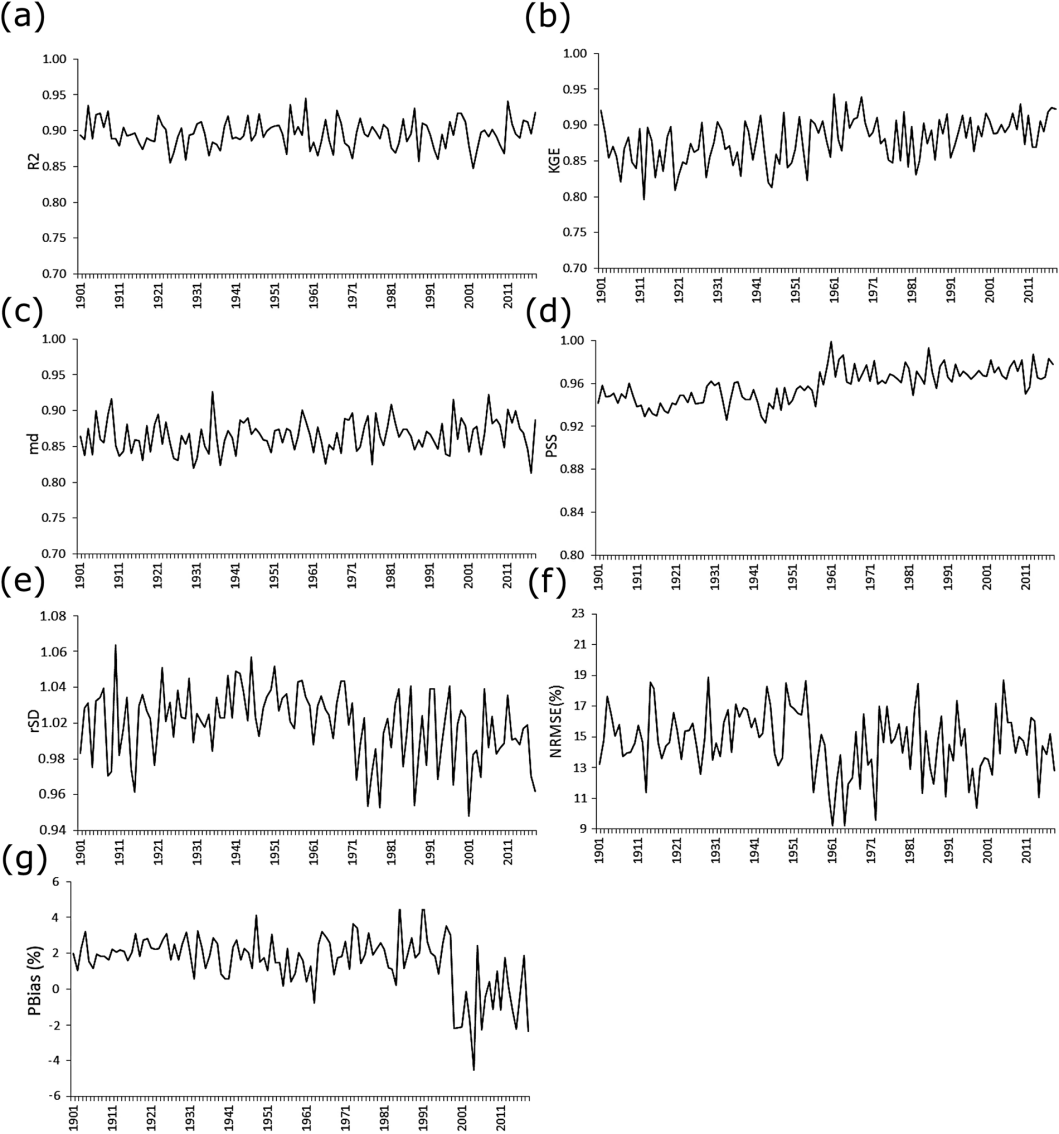
The mean performance of the BDGR. The temporal variability of the mean performance of the BDGR over Bangladesh in terms of (**a**) coefficient of determination (R^2^); (**b**) Kling-Gupta efficiency (KGE); (**c**) Willmott’s modified coefficient of agreement (MD); (**d**) Perkins skill score (PSS); (**e**) ratio of standard deviation (rSD); (**f**) root mean squared error (NRMSE) in %; and (**g**) bias (PBIAS) in %.

The temporal variability in the performance of the BDGR over Bangladesh is shown in Fig. 10. The performance metrics estimated at 322 stations for different years were averaged to show the temporal variable of BDGR performance over Bangladesh for the period 1901‒2018. The results showed the temporal variability of R^2^ in the range of 0.87 to 0.95 and md between 0.84 and 0.93 for the period 1901 to 2018. The KGE showed an increase in performance with time. The mean KGE was 0.85 in the early period (1901–1960) and increased to nearly 0.89 after 1960. Similar improvements were observed in all other metrics after 1960. This is due to the high number of stations used for interpolation after 1960 compared to the early period. It has been mentioned earlier that no data were available at 184 stations until 1960. However, the overall performance of the interpolated rainfall was sufficiently high for the whole period. The PSS was in the range of 0.93 to 0.99, rSD between 0.96 and 1.066, NRMSE between 9 and 19% and PBIAS in the range of −4.5 to 4.3%.

Boxplots of each error metric for different periods were prepared following Lucas, *et al*.^63^ to show the error spread in different periods. Metric values were grouped every five years and presented for 1901–2018 in Supplementary Figure S1. The figure shows no apparent discontinuities in any of the error series.

### Spatial variability

**Fig. 11:**
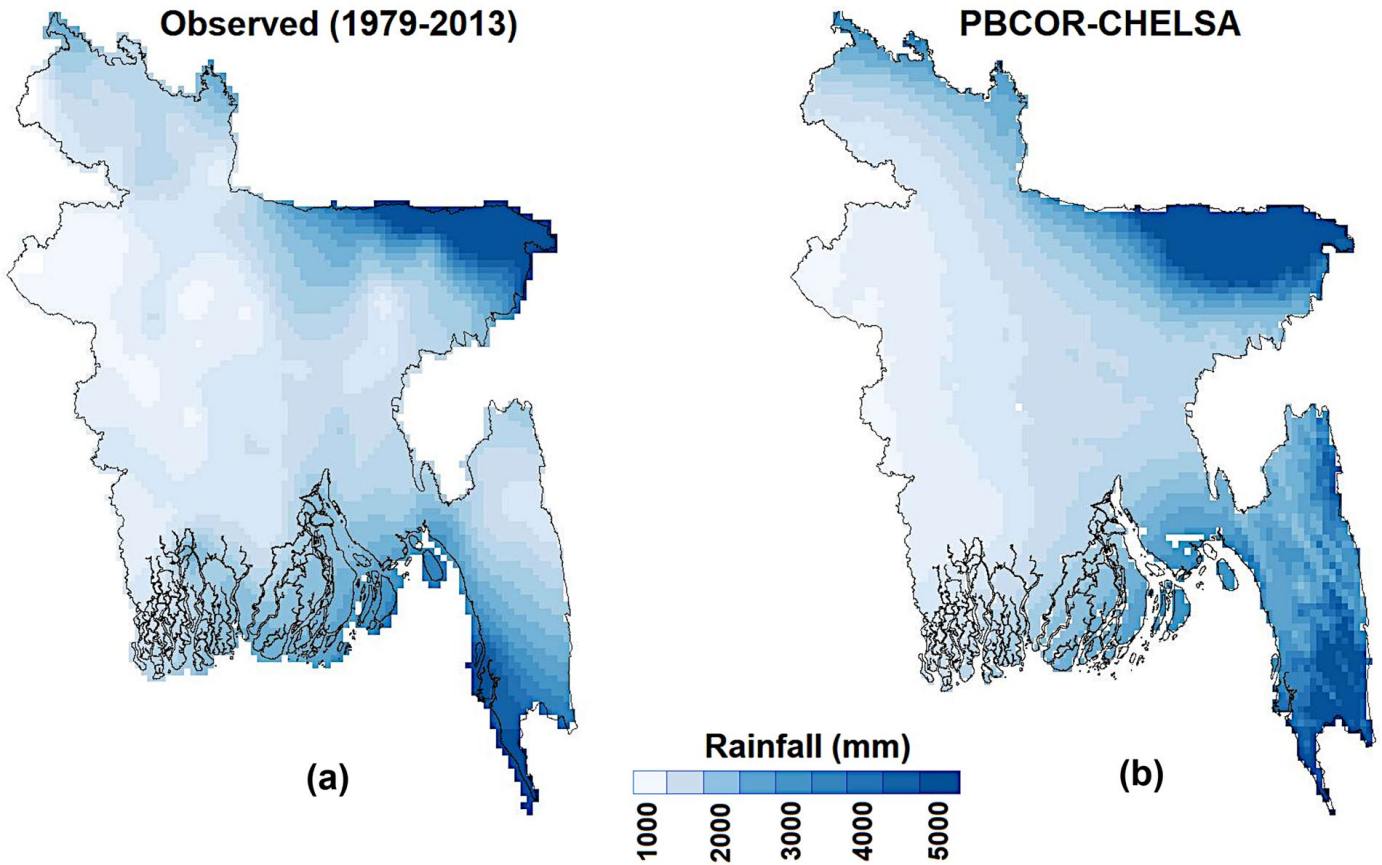
Spatial distribution of annual rainfall. Spatial distribution of annual rainfall: (**a**) BDGR product; (**b**) PBCOR–CHELSA. The 0.01° resolution BDGR data are aggregated to the resolution of PBCOR–CHELSA (0.05°) for comparison. The results show similarity between BDGR and PBCOR–CHELSA annual rainfall climatology. The high and low regions and the transition of rainfall from the high to low zone from west to the east are well captured by the BDGR product.

The annual and monthly BDGR climatologies of BDGR are compared with PBCOR rainfall climatologies to show the performance of BDGR relative to PBCOR. The BDGR and PBCOR CHELSA annual rainfall distributions for 1979–2013 are shown in Fig. 11, and WC and PBCOR CHPclim annual rainfall for the periods 1970–2000 and 1980–2009 are shown in Supplementary Figure S4. In all cases, the 0.01° resolution BDGR data are aggregated to the resolution of PBCOR datasets (0.05°) for comparison. The reason for comparing different datasets is that they all together cover a longer period (1970–2013). The results show that the BDGR annual rainfall climatology was correlated with PBCOR CHELSA by 0.91, WC by 0.89 and CHPclim by 0.88. The high and low regions and the transition of rainfall from the high to low zone from west to the east are well captured by the BDGR product. The PBCOR rainfall climatology is developed with limited station data, and therefore, local heterogeneity in the distribution of rainfall is not visible. However, regional and local heterogeneity was well captured by the BDGR product, indicating its ability to represent the spatial distribution of annual rainfall.

A similar analysis with PBCOR CHELSA was conducted for all months, and their spatial distribution is shown in Supplementary Figure S5. The results showed correlations of BDGR climatology with PBCOR CHELSA between 0.74 and 0.99 for different months.

### BDGR and observed rainfall

The performance of the BDGR was also evaluated to (i) reconstruct temporal patterns, (ii) reproduce seasonal variability, and (ii) estimate trends in different quantiles of observed rainfall. These evaluations were conducted to show the reliability of the BDGR as a substitute for observed rainfall.

**Fig. 12:**
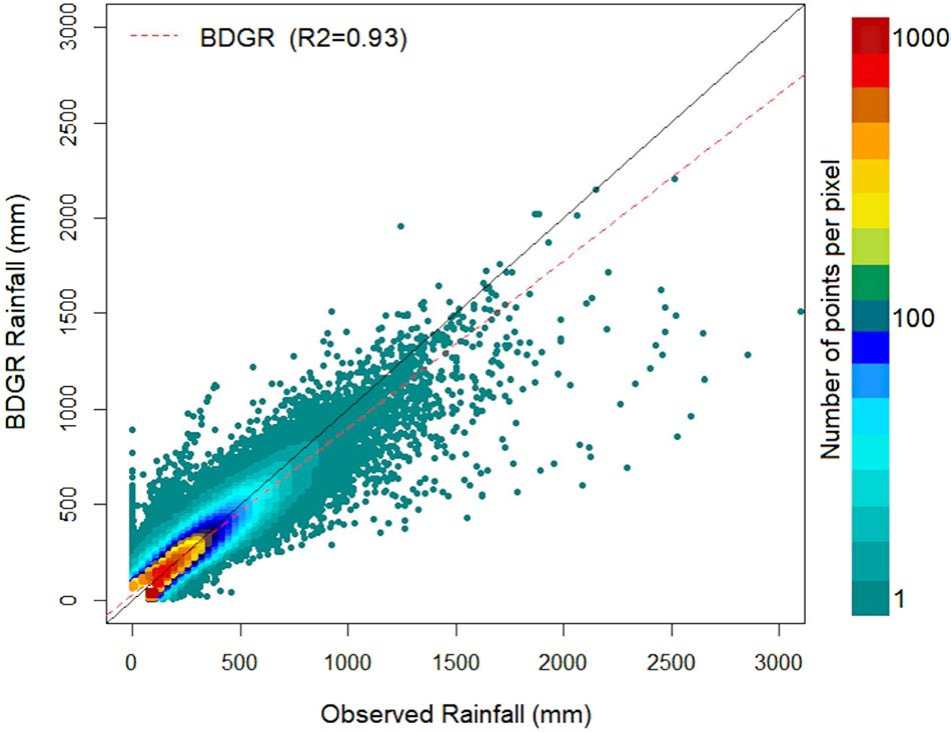
Density scatter plot. Density scatter plot showing the relative performance of the BDGR compared to the observed monthly rainfall (n = 255952). The observed and nearby grid rainfall data for the whole period (1901–2018) are used for comparison. The red colour in the plot indicates dense data, and green shows low density. The best-fit line of the gridded products was presented using a dashed red line. A close correspondence of the regression line to the diagonal (solid black line) indicates better performance of the BDGR product.

The relative performance of the BDGR compared to the observed monthly rainfall is shown using a scatter plot in Fig. 12. The observed and nearby grid rainfall data for the whole period (1901–2018) are used for comparison. Red colour in the plot indicates dense data, and green shows low density. The best-fit line of the gridded products was presented using a dashed red line in the plot. A close correspondence of the regression line to the diagonal (solid black line) indicates better performance of the BDGR product. Figure 9 shows the regression line was closer to the diagonal line. The R^2^ for the BDGR was 0.93, indicating better correspondance of the generated product in replicating rainfall time series across Bangladesh.

**Fig. 13:**
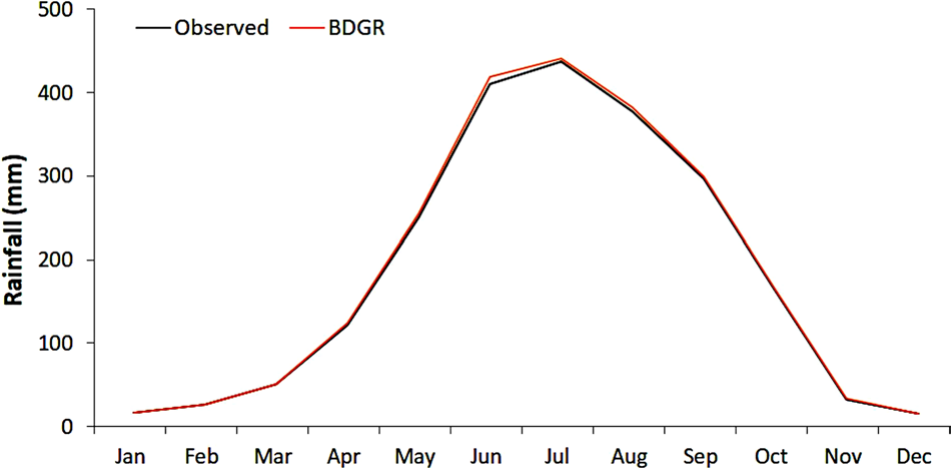
Performance in reconstructing seasonal rainfall variability. Performance of the BDGR in reconstructing seasonal rainfall variability, 1979−2010. All observations were averaged and compared with the average value of all grids of BDGR. The BDGR monthly average rainfall line completely overlapped with the observed data, indicating its ability to reconstruct the seasonal variation in rainfall for the country.

The seasonal performance of the BDGR product is shown in Fig. 13. All observations were averaged and compared with the average value of all grids of BDGR. The comparison was made for each month to demonstrate BDGR’s ability in replicating observed variability of seasonal rainfall. The BDGR monthly average rainfall line completely overlapped with the observed data, indicating its ability to reconstruct the seasonal variation in rainfall for the country.

**Fig. 14:**
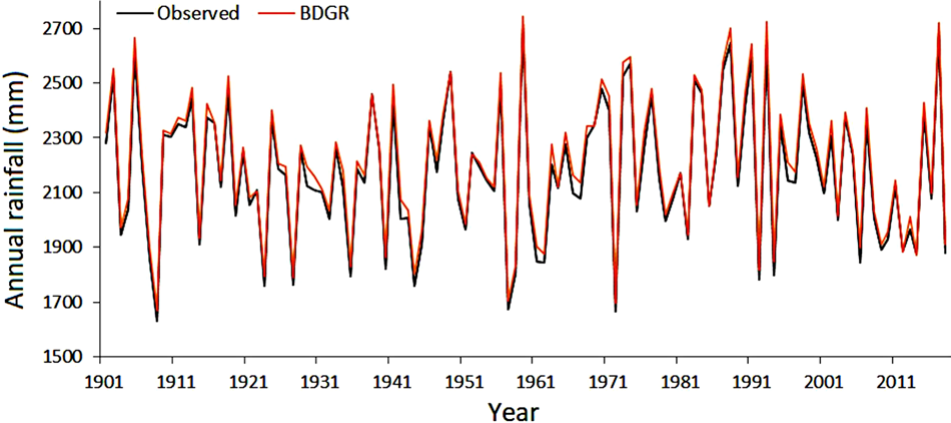
Performance in replicating annual rainfall. Performance of the BDGR product in replicating rainfall in Bangladesh, 1901−2018. The annual rainfall line of the BDGR overlaps with the observed rainfall for the whole study period (i.e., 1901−2018), indicating the ability of newly generated gridded data to estimate the long-term climate variability of the country.

The annual rainfall series provides an estimation of the wet and dry years. In doing so, all observations were first averaged and then converted to annual time series. Likewise, rainfall data of all BDGR grids were average and converted to annual series. The observed and BDGR annual rainfall for the period 1901−2018 are presented in Fig. 14, illustrating that the annual rainfall line of the BDGR overlaps with the observed rainfall for the whole study period (i.e., 1901−2018). Furthermore, this study indicates the ability of newly generated gridded data to estimate the long-term climate variability of the country.

## Data Records

High resolution (0.01°) monthly total rainfall, in mm, for Bangladesh from January 1901 to December 2018, are freely available at 10.6084/m9.figshare.16607912 in NetCDF^64^. The data records spatially, covering land area of Bangladesh. The records will be updated frequently in the upcoming years when more rainfall records will be available. Temporal coverage of the data may be extended in the future when recent observations are accessible.

## Usage Notes

The BDGR product can be used for many applications at various temporal resolutions. The datasets can be used to estimate spatial distribution of rainfall, temporal pattern, seasonality, and the trends more accurately than any other datasets, presently available for the country. Furthermore, this high resolution (e.g., 0.01°) data can be used for localized changes in long–term climate, including the changes in rainfall, and groundwater recharge, along with other scientific and social benefits.

## Supplementary information

Supplementary materials

## Data Availability

A code is written using statistical package (R.4.1) to process data. The code is available online^64^ (10.6084/m9.figshare.16607912).
