# Supplementary material for: Developing a high-resolution gridded rainfall product for Bangladesh during 1901–2018
Source: Sci Data. 2022 Aug 3;9:471. doi: 10.1038/s41597-022-01568-z (PMC9349194; doi:10.1038/s41597-022-01568-z)
Supplement: Supplementary file 1 — Supplementary materials [file 41597_2022_1568_MOESM1_ESM.docx]

**Supplementary Materials**

**
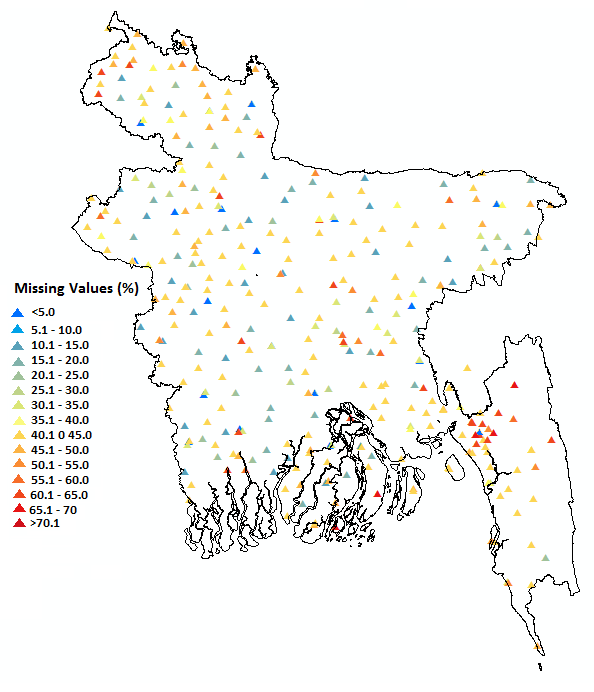
**

Figure S1 Percentage of missing rainfall at different stations in the study area

**
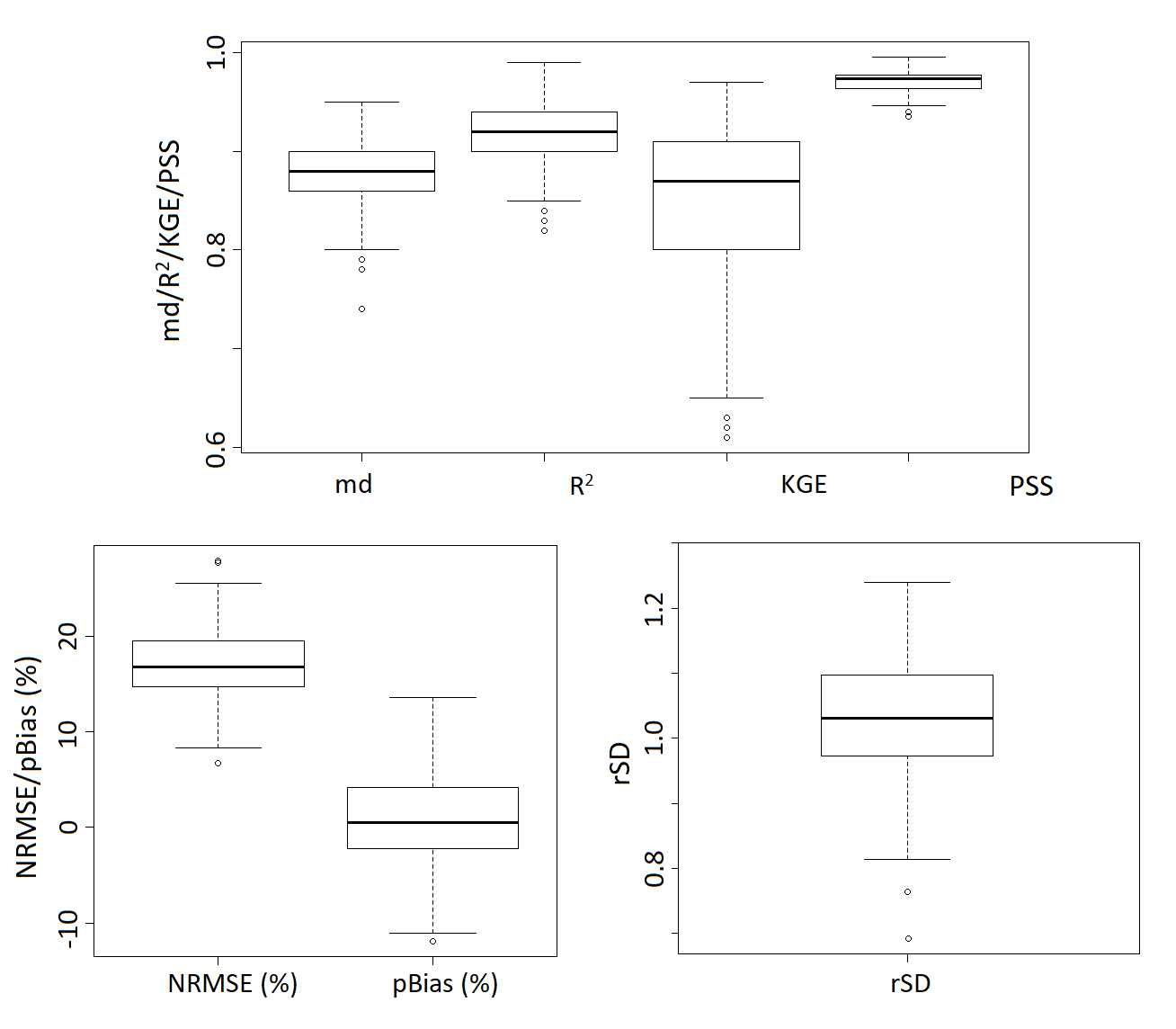
**

Figure S2 Performance of kriging interpolation in reconstructing observed rainfall at all stations during 1901–2018


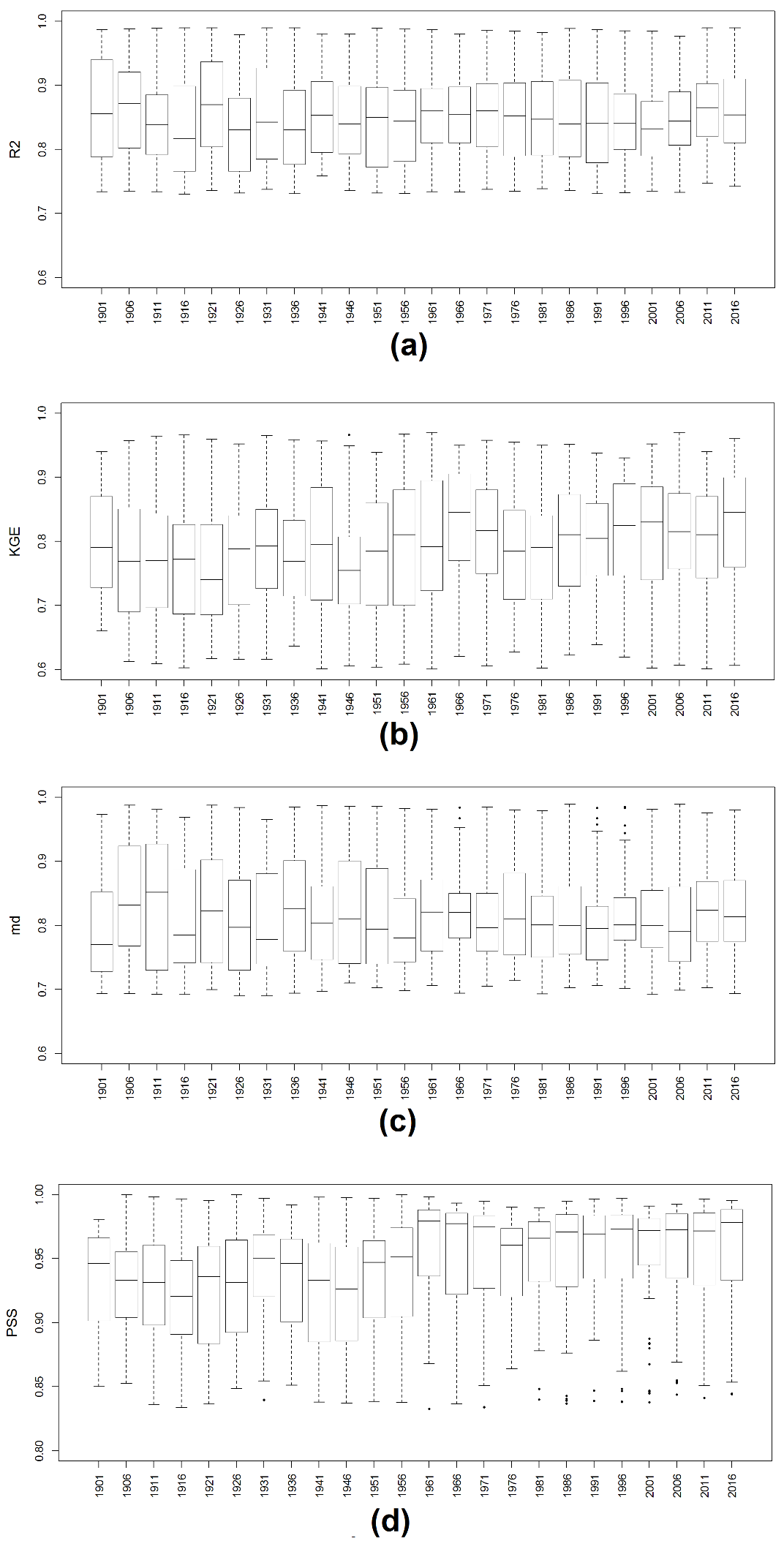


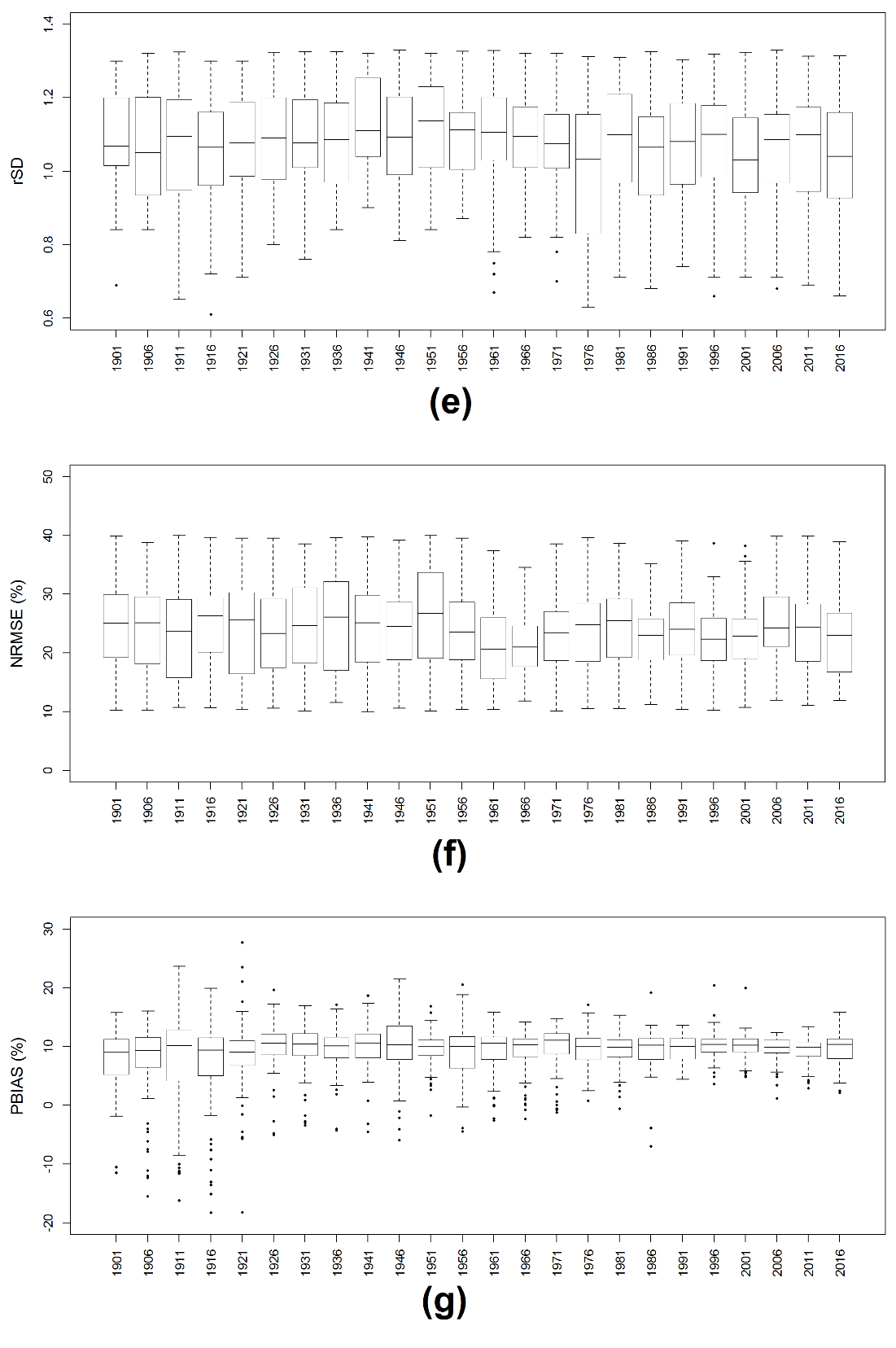


Figure S3 Boxplots of (a) R^2^; (b) KGE; (c) MD; (d) PSS; (e) rSD; (f) NRMSE; and (g) PBIAS. Metric values were grouped every five years and presented for 1901-2018.

**
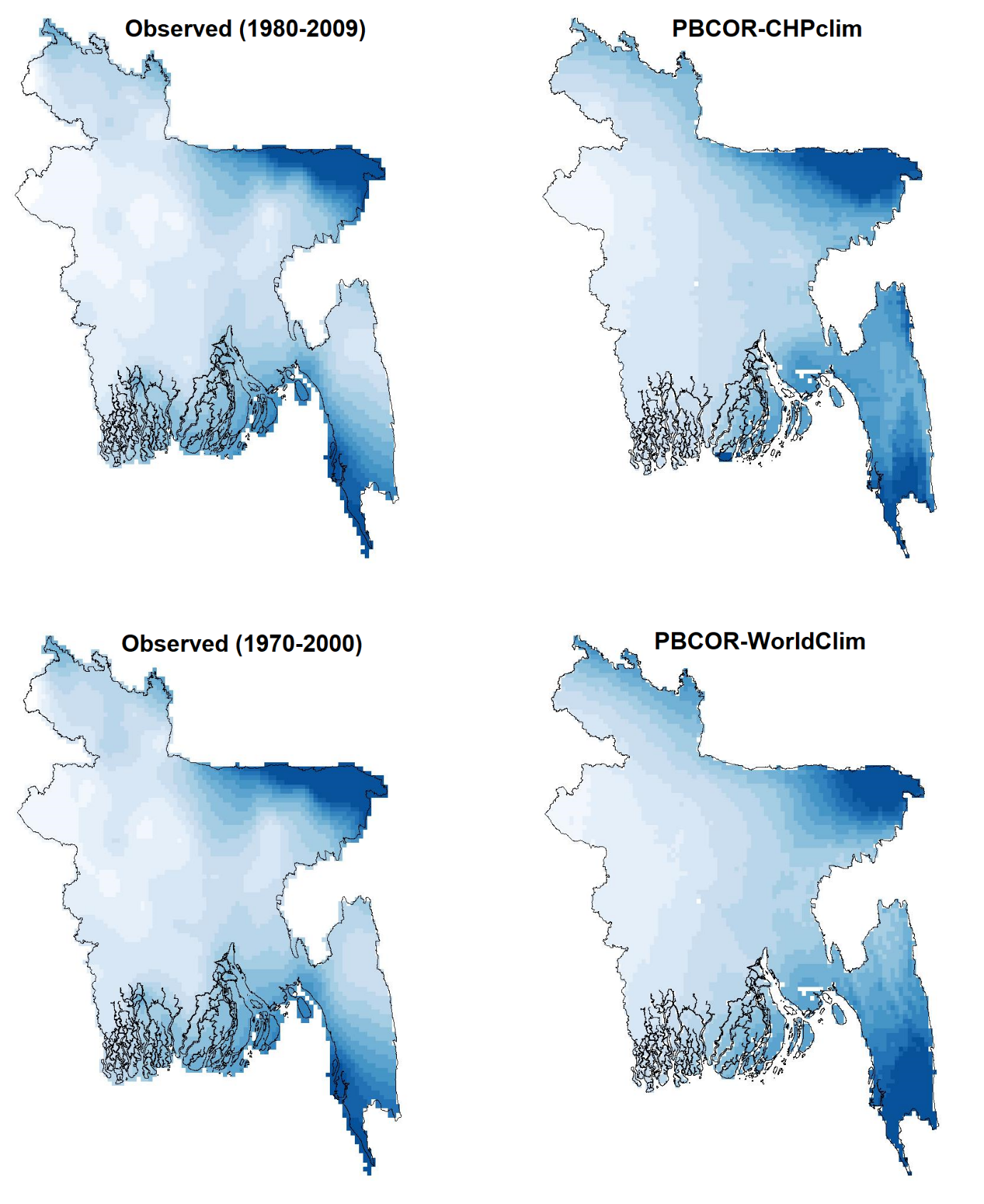
**

Figure S4 Performance of kriging interpolation in reconstructing observed rainfall at all stations during 1901–2018


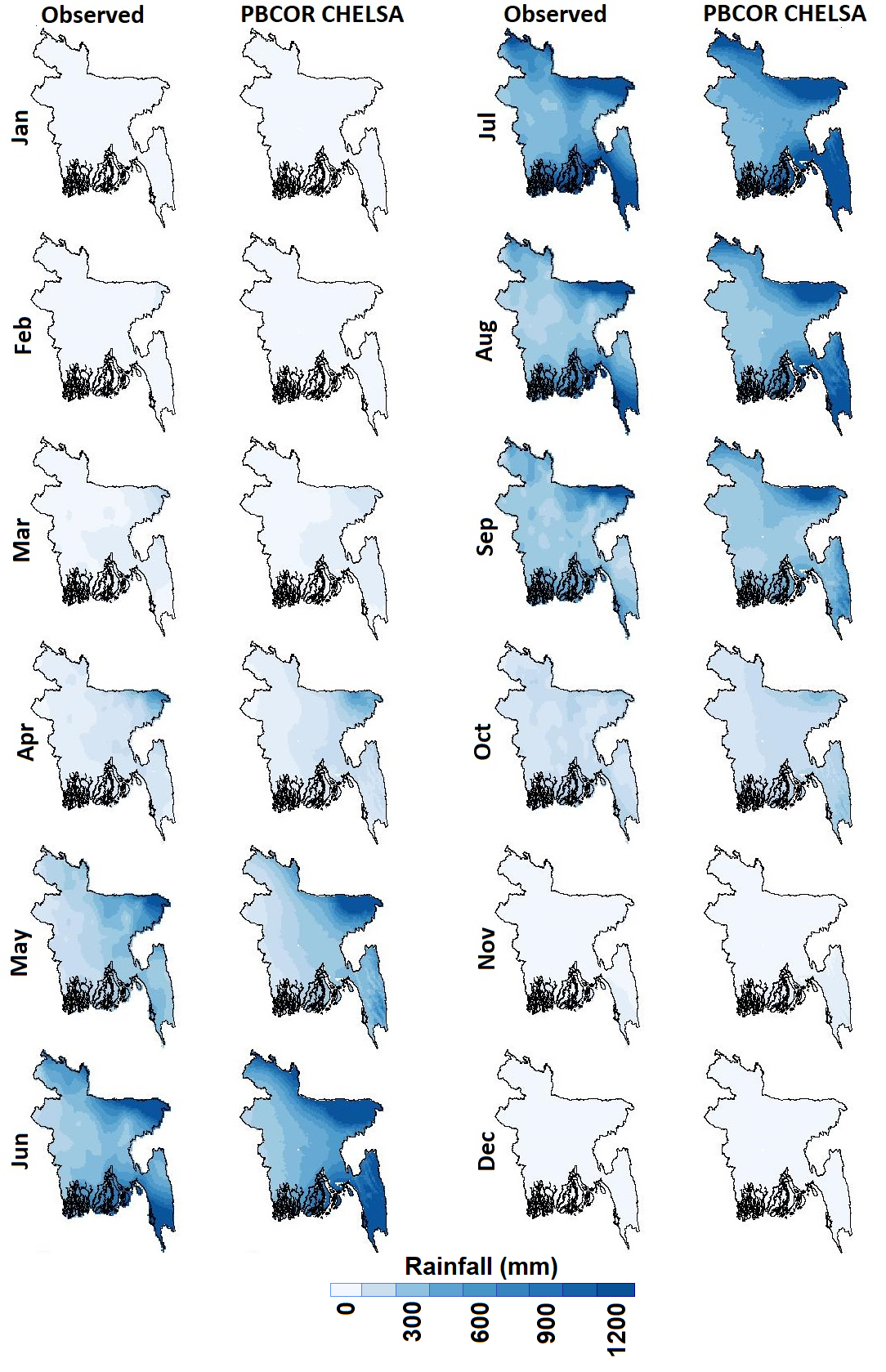


Figure S5 Spatial distribution of the BDGR (left) and PBCOR–CHELSE rainfall during different months
